# Screening Biomarkers for Nerve Injury Using Weighted Gene Co‐Expression Network Analysis and Machine Learning

**DOI:** 10.1002/brb3.71279

**Published:** 2026-02-24

**Authors:** Shuming Cao, Chengyue Yu, Nana Wang, Jianhua Xu, Weiguo Xu

**Affiliations:** ^1^ Clinical School/College of Orthopedics Tianjin Medical University Tianjin China; ^2^ Department of Hand Surgery Tianjin Hospital Tianjin China; ^3^ Tianjin University Tianjin Hospital Tianjin China; ^4^ Department of General Internal Medicine Tianjin Hospital Tianjin China

**Keywords:** biomarker, immune infiltration, nerve injury, nerve regeneration, neuroinflammation, single‐cell RNA sequencing

## Abstract

**Background:**

Nerve injury triggers complex molecular responses involving immune activation and neuronal damage, yet the key regulatory genes and their mechanisms remain poorly understood. Here, we integrated multi‐transcriptomic datasets and machine learning to identify and validate novel biomarkers of nerve injury and elucidate their functional roles.

**Methods:**

The RNA‐seq data and the single‐cell transcriptome data of nerve injury and sham‐surgery samples were sourced from the Gene Expression Omnibus (GEO) database. Weighted gene co‐expression network analysis (WGCNA), differential expression analysis, and three machine learning algorithms were used to identify hub genes associated with nerve injury. The expression patterns and diagnostic value of these hub genes were validated in independent datasets. The correlation between these genes and immune cell infiltration was analyzed using the CIBERSORT algorithm. Finally, single‐cell RNA sequencing (scRNA‐seq) data were used to investigate the cell‐specific expression patterns of the hub genes in neural cells.

**Results:**

Seven nerve injury‐related genes were identified via WGCNA and three machine learning methods, of which *Atf3*, *Bin2*, *Fcgr2b*, and *Ucn* exhibited robust diagnostic performance (AUC > 0.7) across validation cohorts. Functional enrichment implicated these genes in neuroinflammation, neuronal fate commitment, and JAK‐STAT/NF‐κB signaling. Immune infiltration analysis correlated their expression with M2 macrophage polarization and CD4+ T cell depletion, while scRNA‐seq highlighted cell‐specific patterns: *Atf3* and *Ucn* were neuron‐enriched, whereas *Fcgr2b* and *Bin2* predominated in macrophages/NK cells. Moreover, *Fcgr2b* promoted the outgrowth of neurites in PC12 cells.

**Conclusion:**

Our study unveils *Atf3*, *Bin2*, *Fcgr2b*, and *Ucn* as critical nerve injury biomarkers with dual roles in neuroimmune crosstalk, offering novel insights into therapeutic targeting for nerve repair. Moreover, *Fcgr2b* may be involved in neurite outgrowth after nerve injury.

## Graphical abstract

This study identifies *Atf3*, *Bin2*, *Fcgr2b*, and *Ucn* as key diagnostic genes for nerve injury, linking them to neuroinflammation and immune cell dynamics. *Fcgr2b* was further shown to functionally promote neurite outgrowth.

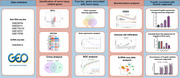

## 1 Introduction

Nerve injury is a significant and well‐recognized subject in the medical field, with its incidence rate showing an annual increasing trend, which imposes a substantial burden on patients' quality of life and the socio‐economic landscape (Girach et al. 2019; Harhaus et al. 2024; Magneli and Axenhus 2024). Nerve injuries are categorized into two major types: central nerve injuries and peripheral nerve injuries. Peripheral nerve injuries, as common neurological disorders, can arise from various factors such as trauma and disease, leading to symptoms including motor, sensory, and autonomic nerve dysfunction in patients (Chu et al. 2022). Central nervous system injuries, such as spinal cord injuries and brain injuries, are primarily caused by severe trauma, cerebrovascular diseases, or tumors, often resulting in more severe consequences, including paralysis and cognitive disorders (Gao et al. 2024; Montoto‐Marques et al. 2025; Tian et al. 2023). Despite extensive research on nerve injuries, significant breakthroughs in therapeutic advancements have yet to be achieved (Hussain et al. 2020; Lopes et al. 2022). Therefore, conducting in‐depth research into the mechanisms of nerve injury is particularly important for identifying potential therapeutic targets.

The pathological features of nerve injury are characterized by their diversity and complexity, encompassing a series of changes at both the cellular and overall tissue levels (Dong et al. 2025). In the case of peripheral nerve injury, the initial response is Wallerian degeneration (Tian et al. 2025). During this process, the distal axon and myelin segments undergo a series of degenerative changes, resulting in the breakdown and clearance of damaged tissue (Tian et al. 2025). This process is accompanied by an inflammatory response involving macrophages and Schwann cells, which play a crucial role in debris clearance and in preparing the environment for potential regeneration (Rotshenker 2011). However, the regenerative capacity of peripheral nerves is limited by various factors, including the distance between the injury site and the target tissue, the presence of scar tissue, and the availability of appropriate growth factors and cellular support (Li et al. 2020). Collectively, these factors determine the efficiency of neural regeneration and the extent of functional recovery. Furthermore, the central nervous system (CNS) has a highly restricted ability to spontaneously regenerate after injury. The pathological hallmarks of central nervous system injury include primary mechanical damage, followed by a complex cascade of secondary injuries (Alizadeh et al. 2019; Kvistad et al. 2024). This cascade involves excitotoxicity, oxidative stress, inflammation, and apoptosis—processes that may exacerbate tissue damage and lead to extensive neuronal death and glial scar formation (Anjum et al. 2020). The glial scar, primarily composed of astrocytes and microglia, creates a physical and biochemical barrier that further inhibits axonal regeneration and functional recovery (Gao et al. 2013; Manrique‐Castano et al. 2024).

In light of the numerous challenges posed by neural damage, neural regeneration has emerged as a focal point of research. This complex and delicate biological process involves several critical stages: the survival of damaged nerve cells, the extension of axons, the remodeling of myelin sheaths, and the re‐establishment of effective connections with surrounding neural tissues and target organs (Pandey and Mudgal 2022). However, due to limitations imposed by human physiological mechanisms and various external environmental factors, the natural progression of neural regeneration often fails to achieve an ideal state, resulting in an incomplete restoration of damaged nerves to normal functional levels (Lin et al. 2025; Tang 2020; Tataranu and Rizea 2025). This situation underscores the urgent need for in‐depth research into the mechanisms of nerve injury and neural regeneration, and the exploration of reliable therapeutic targets.

Although numerous biomarkers associated with nerve injury have been identified in current research, their clinical translation and application remain significantly limited. Many existing biomarkers lack sufficient diagnostic specificity to accurately distinguish between different stages and types of nerve injury (Li et al. 2024). Their cell‐type‐specific expression patterns and functional mechanisms within neuro‐immune interactions are still poorly understood. More importantly, individual biomarkers often fail to comprehensively reflect the complex pathophysiology of neural damage, while systematic screening and validation studies for combinatorial biomarker panels remain notably scarce.

In this study, we employed an integrated bioinformatics approach combined with multiple machine learning algorithms to perform comparative analysis of gene expression profiles between nerve injury and sham surgery groups. By identifying key differentially expressed genes, we elucidated their potential mechanisms in nerve injury pathogenesis and preliminarily investigated their associations with immune cells as well as their roles in nerve regeneration. This study does not merely search for differentially expressed genes in isolation; instead, it integrates WGCNA, multiple machine learning algorithms, immune infiltration analysis, and single‐cell sequencing to systematically screen and validate biomarkers with high diagnostic value and clear functional mechanisms, particularly emphasizing their dual roles in neuro‐immune interactions.

## 2 Materials and Methods

### 2.1 Data Acquisition

The RNA‐seq datasets (GSE236754, GSE202111, GSE172133, GSE102721, GSE175760) and the single‐cell transcriptome data (GSE255014) were sourced from the Gene Expression Omnibus (GEO) database (https://www.ncbi.nlm.nih.gov/geo/). All details corresponding to individual datasets are summarized in Table 1. Based on the rat genome annotation file provided by the NCBI website (https://www.ncbi.nlm.nih.gov/datasets/genome/GCF_036323735.1/), the count data from the GSE236754, GSE202111, and GSE172133 datasets will be converted into Transcripts Per Million (TPM) data. After addressing the batch effects using the “sva” package (version 3.50.0) in R, the datasets GSE236754, GSE202111, and GSE172133 were combined and utilized as a training cohort. The optimal number of surrogate variables (n.sv = 2) was determined using the num.sv function with the “be” method. These two surrogate variables were included alongside the primary variable of interest in the ComBat model to preserve biological signals during batch effect adjustment. The GSE102721, GSE175760, GSE256472, GSE24982, and GSE220261 datasets served as independent validation sets.

**TABLE 1:** Information on all the datasets in this study.

Datasets	Nerve injury	Sham‐surgery	Experiment type	Platform
GSE236754	16	16	High throughput sequencing	Illumina HiSeq 4000
GSE202111	6	3	High throughput sequencing	Illumina HiSeq 2000
GSE172133	15	3	High throughput sequencing	Illumina HiSeq 2500
GSE102721	9	3	High throughput sequencing	Illumina HiSeq 4000
GSE175760	15	3	High throughput sequencing	Illumina HiSeq 4000
GSE255014	2	2	High throughput sequencing	Illumina NovaSeq 6000

### 2.2 Weighted Gene Co‐Expression Network Analysis (WGCNA)

WGCNA (Langfelder and Horvath 2008) was utilized to identify module genes associated with nerve injury and investigate the correlation between nerve injury and module genes. Based on the absolute median deviation of gene expression values, the top 50% of genes were selected for WGCNA using the WGCNA package (version 1.72‐5) in R. The Pearson correlation coefficient between each pair of genes was calculated, and an appropriate soft threshold *β* was selected to ensure that the constructed network better met the criteria of a scale‐free network. The gene network was constructed using a one‐step method, the adjacency matrix was transformed into a Topological Overlap Matrix (TOM), and a hierarchical clustering tree of genes was generated using hierarchical clustering. Gene significance and module significance were calculated to measure the significance of genes and traits, the associations between modules were analyzed, and the gene lists within modules were obtained.

### 2.3 Differential Expression Analysis and Enrichment Analysis

Differential expression analysis was performed using the “limma” (version 3.56.2) package (Ritchie et al. 2015) in R. Differentially expressed genes (DEGs) between the nerve injury group and Sham‐surgery group were screened based on |Log2FC| > 0.5 and *p* value < 0.05. Next, DEGs were subjected to Gene Ontology (GO) enrichment analysis, which encompassed Biological Processes (BP), Molecular Functions (MF), and Cellular Components (CC). Additionally, Kyoto Encyclopedia of Genes and Genomes (KEGG) enrichment analysis was performed using the “clusterProfiler” package (version 4.8.3) (Yu et al. 2012) in R.

### 2.4 Gene Set Enrichment Analysis (GSEA)

Based on the median expression level of the target gene, samples were classified into two groups: high‐expression group and low‐expression group. Subsequently, the DEGs between two groups were identified and used for GSEA. The significantly enriched pathways were screened using *p*.adjust < 0.05. The GO biological functions of the genes were analyzed using the ClueGO plugin (version 2.5.10) of Cytoscape software (version 3.10.2). The analysis was conducted with parameter screening set to a *p* value threshold of 0.7.

### 2.5 Multiple Machine Learning Algorithms

LASSO logistic regression was performed using the R package glmnet (v4.1‐10) with a binomial family setting. The optimal penalty parameter (λ) was determined via 10‐fold cross‐validation, selecting the value (lambda.min) that minimized the misclassification error. Features with non‐zero coefficients at this λ were retained as key genes.

Random Forest (RF) analysis was implemented with the randomForest package (v4.7‐1.2). An initial model was built with 1000 trees (ntree = 1000) and 2 randomly sampled features per split (mtry = 2). The final model used 500 trees, optimized based on out‐of‐bag (OOB) error. Feature importance was evaluated by the mean decrease in Gini index, with the top 13 genes (optiontrees = 13) exhibiting the lowest error rate selected.

Support Vector Machine Recursive Feature Elimination (SVM‐RFE) was carried out using the msvmRFE algorithm with 10‐fold cross‐validation. A halving strategy was applied when features exceeded 100 (halve.above = 100). A linear kernel with a default cost parameter (C = 1) was used, and the optimal feature subset size was determined as 9 based on the error profile.

### 2.6 Prediction of miRNA‐mRNA Interactions

Potential miRNAs targeting the key genes (Atf3, Bin2, Fcgr2b, and Ucn) were predicted using the miRWalk database, with a stringent binding probability threshold of *p* value > 0.9.

### 2.7 Analysis at the Single‑Cell Level

The “Seurat” package [version 5.1.0] in R was used to perform downstream analysis on the scRNA data of the GSE255014 gene set. To ensure the quality of our RNA sequencing data, data preprocessing was performed using the subset function. To filter out low‐quality cells, strict quality filter criteria were applied to each cell (nFeature_RNA > 200, nFeature_RNA < 5000, percent.mt < 20%). The data were normalized and scaled using the “LogNormalize” method from the NormalizeData function, followed by the identification of principal components through Principal Component Analysis (PCA). Batch effects were mitigated using the “Harmony” function. Subsequently, the cells were clustered employing the FindNeighbors and FindClusters functions (resolution = 0.5). The cell clusters were annotated utilizing the SingleR [version 2.4.1] package. Finally, the results of the annotation and the expression patterns of key genes were visualized using the RunUMAP function.

### 2.8 Immune Cell Infiltration

The relative proportions of 22 immune cell types in the samples were calculated using the CIBERSORT software (Newman et al. 2015). Using the gene expression matrix, the deconvolution algorithm was applied with a predefined set of 547 barcode genes to characterize the composition of immune‐infiltrating cells. The total estimated proportions of all immune cell types in each sample sum to one. In addition, the abundance of 22 specific immune cell types was determined using the ssGSEA algorithm.

### 2.9 Cell Culture and Transfection

PC12 cells were obtained from IMMOCELL (IM‐R014) and cultured in RPMI‐1640 medium (IMMOCELL, PM150110) supplemented with 5% fetal bovine serum (FBS) (Procell, PM164210), 10% horse serum (IMMOCELL, IMC‐108), and 1% penicillin‐streptomycin (P/S) (Procell, P1400) at 37°C in a humidified atmosphere containing 5% CO2. The Fcgr2b siRNAs (si‐Fcgr2b‐1, si‐Fcgr2b‐2, si‐Fcgr2b‐3) and si‐negative control (NC) were sourced from TSINGKE (Table 2). For the transfection of Fcgr2b siRNA plasmids into PC12 cells, Lipofectamine 2000 (Invitrogen, Carlsbad, CA) was utilized in accordance with the manufacturer's instructions. Subsequently, the cells were stimulated with 100 ng/mL nerve growth factor (NGF) (Thermo, 450‐34‐20UG) for 0, 1, 3, and 5 days, and photographed using an inverted microscope (Olympus, IMT‐2). The length of neurite PC12 cells was measured using ImageJ.

**TABLE 2:** The sequences of si‐Fcgr2b and siRNA‐NC.

Name		Sequence (5′–3′)
Si‐Fcgr2b‐1	sense	GACUUUGUACCAUAUGCUA(dT)(dT)
	antisense	UAGCAUAUGGUACAAAGUC(dT)(dT)
Si‐Fcgr2b ‐2	sense	CAUUGUUAUUAUCCUGCUA(dT)(dT)
	antisense	UACCAGGAUAAUAACAAUG(dT)(dT)
Si‐Fcgr2b ‐3	sense	GCUACUCCAGACCUCUCAA(dT)(dT)
	antisense	UUGAGAGGUCUGGAGUAGC(dT)(dT)
SiRNA‐NC	sense	UUCUCCGACAGUGUCACGU (dT)(dT)
	antisense	ACGUGACACUGUCGGAGAA (dT)(dT)

### 2.10 RT‐qPCR Assay

Total RNA was isolated from the cells utilizing TriQuick Reagent (Solarbio, R1100). For reverse transcription, the Evo M‐MLV RT Premix (AG11706‐S, Accurate Biotechnology [Hunan] Co., Ltd.) was employed. PCR amplification was conducted using the 2×SuperStar Universal SYBR Master Mix (CW3360, CWBIO), while quantitative real‐time PCR was executed with a PCR instrument (Shanghai Hongshi Medical Technology Co., Ltd, SLAN‐96S). The sequences of the primers can be found in Table 3. The thermal cycling program included 40 cycles of 95°C for 30 s, followed by 95°C for 10 s, and 60°C for 30 s. The internal control selected was β‐actin, and mRNA expression levels were assessed using the 2^−ΔΔCT^ method (Jiang et al. 2020).

**TABLE 3:** Primer sequences for qPCR.

Genes	Forward primer (5′–3′)	Reverse primer (5′–3′)
Fcgr2b	CTGGTGAAGGTGCTGAAGAA	GCTGTGGTAGGTGTTGCTGT
NF200	CAGCAGATCAAGGAGCTGTG	TGGCATCTTCTCCAGCTTCT
β‐actin	GGCTGTATTCCCCTCCATCG	CCAGTTGGTAACAATGCCATGT

### 2.11 Western Blot Assay

Proteins were isolated from cells with the use of a homogenizer in RIPA buffer (R0010, Solarbio). The concentrations of proteins were measured employing the BCA protein assay kit (CW0014S, CWBIO). The western blotting procedure was conducted as per previously established methodologies (Qian et al. 2020). The primary antibodies utilized included β‐actin (Proteintech, 1:10000), Fcgr2b (Invitrogen, 1:1000, MA5‐47232), and NF200 (Proteintech, 60331‐1‐Ig, 1:10000). For secondary detection, HPR‐Labeled Goat Anti‐Rabbit IgG (H + L) (Affinity Purified) (Beijing Zhongshan Jinqiao Biological Technology Co., Ltd., ZB‐2301) and HPR‐Labeled Goat Anti‐Rabbit IgG (H + L) (Affinity Purified) (Beijing Zhongshan Jinqiao Biological Technology Co., Ltd., ZB‐2305) were employed. *β*‐actin functioned as the internal control. The detection of protein bands was carried out using a fully automated chemiluminescence image analysis system (Clinx, Chemi6000). Western blot band intensities were measured with ImageJ software by quantifying grayscale values. The relative expression level of the target protein is presented as fold change over the control, which was calculated as: (Target protein/Internal control) experimental group/(Target protein/Internal control) control group.

### 2.12 Statistical Analysis

The Wilcoxon rank‐sum test was utilized to compare gene expression differences across various groups. Pearson correlation analysis was performed using the R programming language. A *p* value < 0.05 was deemed statistically significant. All statistical analyses mentioned were conducted using R software version 4.4.1.

## 3 Results

### 3.1 Identification of Nerve Injury‐Related Genes via WGCNA

**FIGURE 1:**
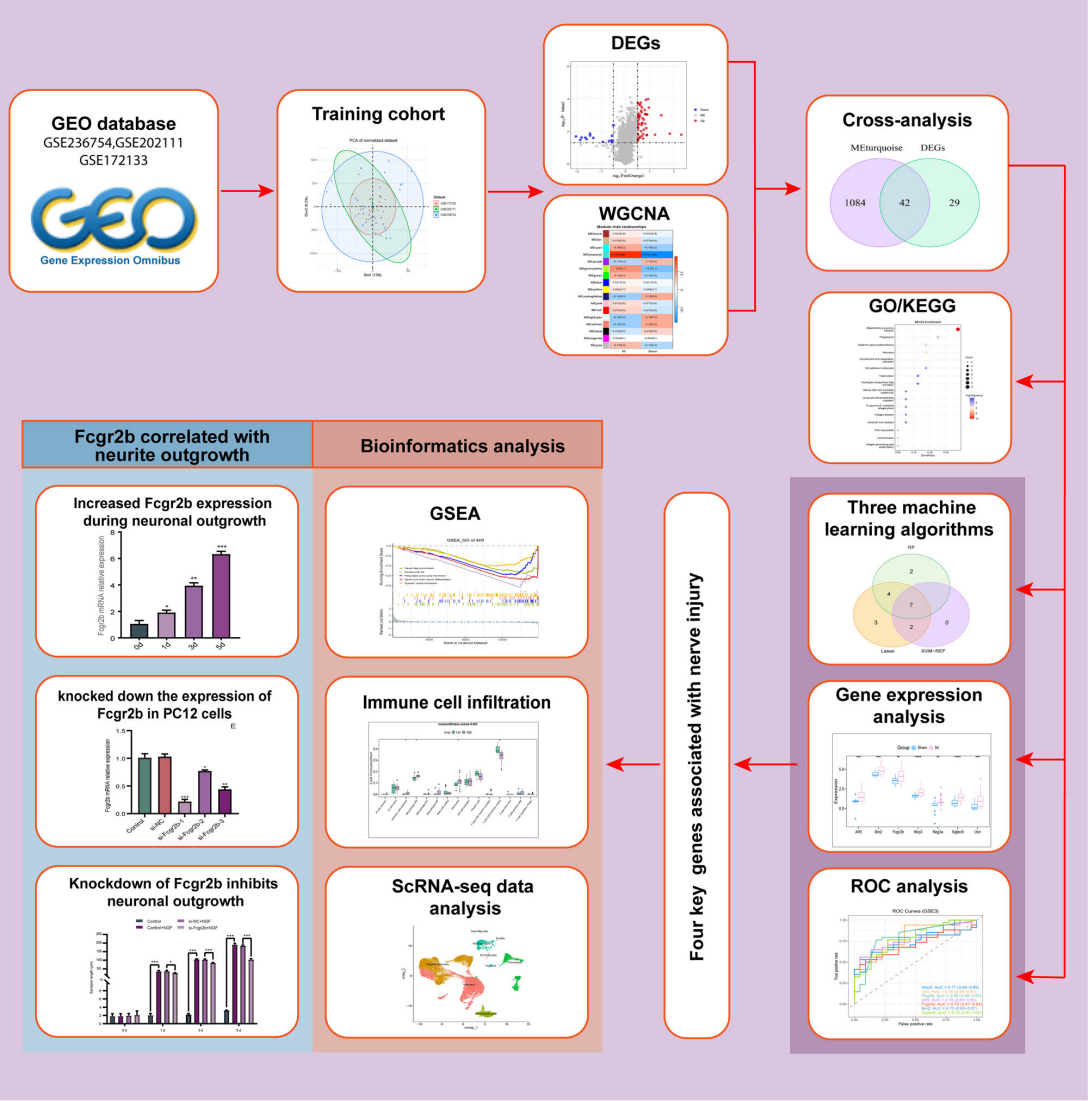
The flowchart of this study.

**FIGURE 2:**
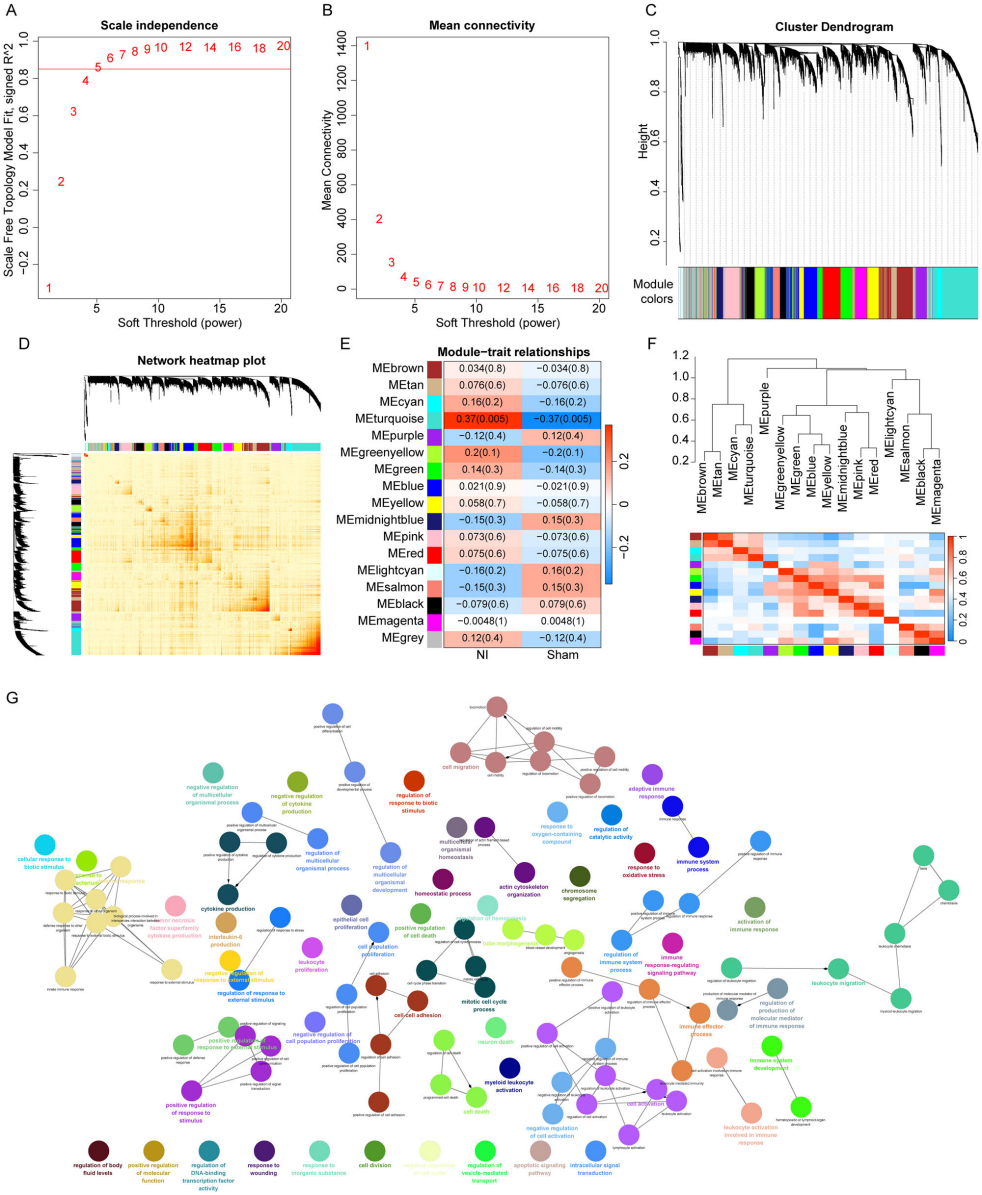
Identification of nerve injury‐related genes via weighted gene co‐expression network analysis (WGCNA). (A)–(B) Analysis of the scale‐free fitting index for soft threshold powers (β) and the mean connectivity for soft threshold powers. (C) The gene co‐expression network and module results. The upper section displays the hierarchical clustering dendrogram of genes, while the lower section represents the gene modules. Each module is identified by a specific color, with the grey module comprising genes that could not be grouped into other modules. (D) Gene co‐expression network heatmap. The horizontal and vertical axes of the heatmap represent the modules to which each gene belongs, and the heatmap illustrates the correlation between each gene, with darker colors indicating stronger correlations. (E) Heatmap of the Pearson correlation coefficient (PCC) between module eigengenes (MEs) and nerve injury. The color bar displayed on the far right illustrates the correlation range, where red signifies a positive correlation and blue denotes a negative correlation. The intensity of the color reflects the strength of the correlation. (F) The correlation among gene modules. The color bar displayed on the far right illustrates the correlation range, where red signifies a positive correlation and blue denotes a negative correlation. The intensity of the color reflects the strength of the correlation. (G) Network diagram of ClueGO enrichment analysis.

The flowchart of the analysis steps is shown in Figure 1. First, we merged the GSE236754, GSE202111, and GSE172133 datasets to obtain a total of 34 nerve injury samples and 22 sham surgery samples, and named it the training cohort. Subsequently, we eliminated the batch effect present in the data. Following the normalization of the dataset, the clustering distribution appeared uniform, which indicates the reliability of the data (Figure S1A–D). To identify the gene modules associated with nerve injury, we constructed a WGCNA using samples from the training cohort. We selected a soft threshold of *β* = 5 (Figure 2A,B) as the optimal value and identified 16 gene modules (Figure 2C,D). Among these modules, the turquoise module showed a significant correlation with nerve injury (Figure 2E,F), encompassing a total of 1126 genes (Table S1). These 1126 genes were significantly enriched in pathways related to immune response activation, regulation of responses to biotic stimuli, and neuronal death (Figure 2G, Table S1).

### 3.2 Identification of DEGs Between Nerve Injury and Sham Surgery Groups

**FIGURE 3:**
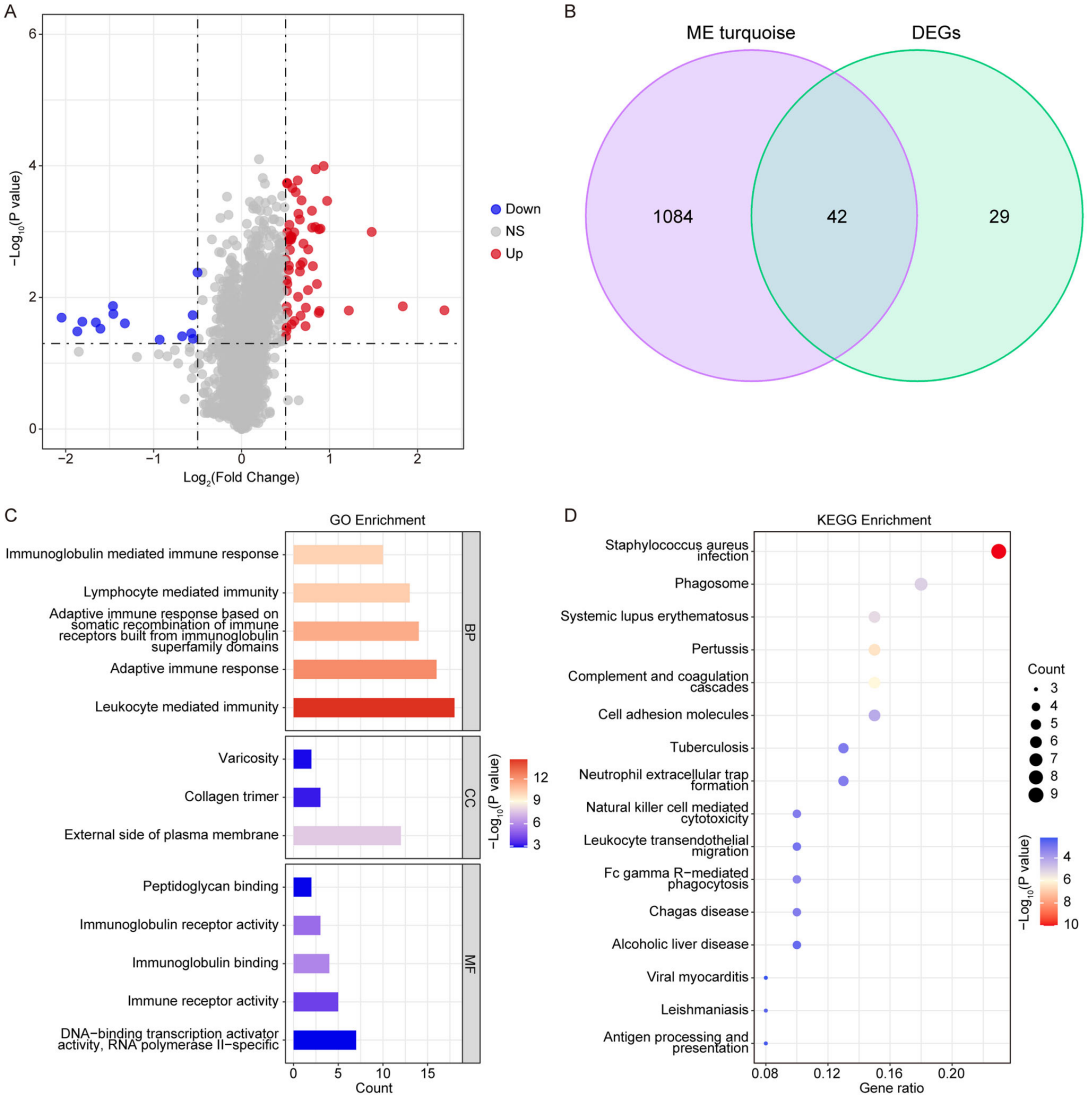
Identification of DEGs between nerve injury and sham surgery groups and enrichment analysis. (A) Differentially expressed genes between the nerve injury (*n* = 34) and sham surgery (*n* = 22) groups in the training cohort. (B) Cross‐analysis between Differentially expressed genes and turquoise module‐related genes from WGCNA. C. The results of Gene Ontology enrichment analysis. (D) The results of Kyoto Encyclopedia of Genes and Genome enrichment analysis. BP, biological processes; MF, molecular functions; CC, cellular components.

In the training cohort, a total of 71 DEGs were identified between the nerve injury and sham surgery groups, comprising 14 downregulated genes and 57 upregulated genes (Figure 3A, Table S2; nerve injury vs. sham surgery, *p* < 0.05). Consequently, 42 overlapping genes were identified by intersecting the turquoise module‐related genes from WGCNA with DEGs (Figure 3B, Table S2). These genes were considered to be candidate nerve injury‐related genes. GO enrichment analysis showed that these 42 genes were highly enriched in immune‐related biological processes, such as leukocyte‐mediated immunity, adaptive immune response, immunoglobulin receptor activity, and immune receptor activity (Figure 3C, Table S3). KEGG revealed that these 42 genes were significantly correlated with *Staphylococcus aureus* infection, pertussis, and complement and coagulation cascades pathways (Figure 3D, Table S3). These findings suggest that genes associated with nerve injury may be closely related to immune function.

### 3.3 Machine Learning to Identify Important Nerve Injury‐Related Genes

**FIGURE 4:**
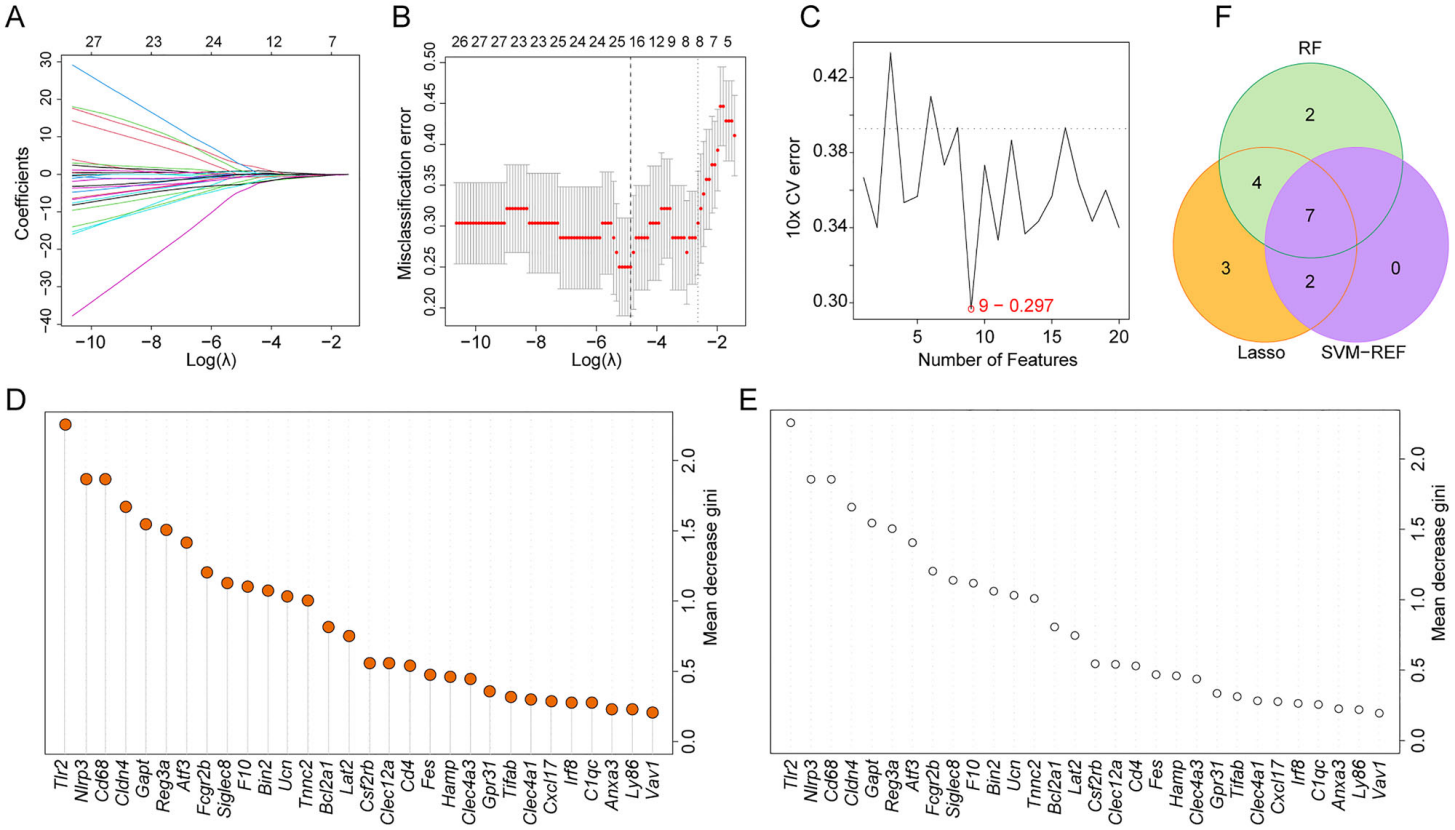
Machine learning to identify important nerve injury‐related genes. (A) The Lasso coefficient path plot illustrates the trajectory of coefficient changes for each gene. The vertical axis represents the coefficient values, while the lower horizontal axis displays the logarithm of the penalty coefficient, log λ. The upper horizontal axis indicates the number of non‐zero coefficients present in the model at each corresponding point. (B) The 10‐fold cross‐validation curve of the Lasso model. The lower horizontal axis represents the logarithm of the penalty coefficient log λ, the vertical axis represents the misclassification error rate, and the upper horizontal axis indicates the number of non‐zero coefficients in the model at that point. (C) The 10‐fold cross‐validation error rate graph of the SVM‐RFE model. The vertical axis represents the error rate, and the horizontal axis represents the number of feature genes corresponding to that error rate. The red circle indicates the minimum error rate point, where there are 9 feature genes at that error rate. (D) Error rate plot of the Random Forest model. The horizontal axis represents the number of random classification trees, and the vertical axis represents the test error rate. (E) RF Model Gene Importance Coefficient Diagram. The vertical axis represents the feature genes, and the horizontal axis represents the importance coefficients of the feature genes. (F) The Venn diagram illustrates hub genes screened by three algorithms.

To enhance the accuracy of identifying important nerve injury‐related genes, we employed three machine learning algorithms: LASSO, SVM‐RFE, and RF for gene screening. Utilizing lambda as a parameter, LASSO identified 16 feature genes (Figure 4A,B). The SVM‐RFE algorithm identified nine genes (Figure 4C), while RF pinpointed 13 genes as potential nerve injury biomarkers (Figure 4D,E, optiontrees = 13). Ultimately, seven overlapping genes—*Nlrp3*, *Ucn*, *Reg3a*, *Atf3*, *Fcgr2b*, *Bin2*, and *Siglec8*—were obtained (Figure 4F). The results of the three machine learning algorithms used for identifying seven nerve injury‐related genes are presented in Table S4.

### 3.4 *Atf3*, *Bin2*, *Fcgr2b*, and *Ucn* as Key Genes Associated With Nerve Injury

**FIGURE 5:**
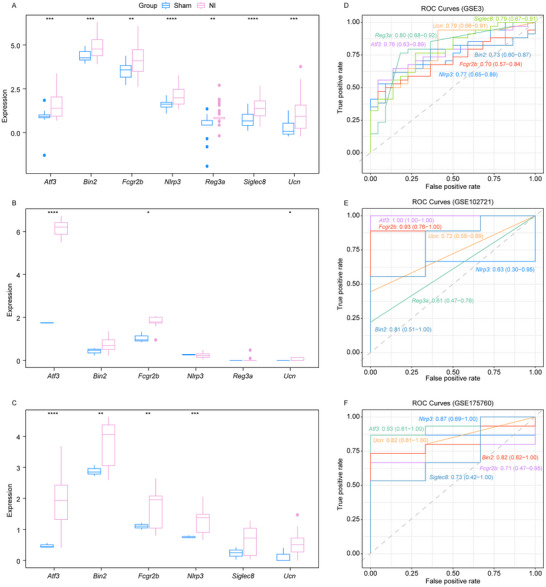
*Atf3*, *Bin2*, *Fcgr2b*, and *Ucn* as key genes associated with nerve injury. (A) The expression of *Atf3* (^***^ *p* = 0.00018), *Bin2* (^***^ *p* = 0.00028), *Fcgr2b* (^**^ *p* = 0.00121), *Nlrp3* (^****^ *p* = 3.1e‐05), *Reg3a* (^**^ *p* = 0.00324), *Siglec8* (^****^ *p* = 7.4e‐05), and *Ucn* (^***^ *p* = 0.00011) in nerve injury (*n* = 34) and sham (*n* = 22) samples in the training cohort. (B) The expression of *Atf3* (^****^ *p* = 4.6e‐10), *Bin2*, *Fcgr2b* (^*^ *p* = 0.012), *Nlrp3*, *Reg3a*, and *Ucn* (^*^ *p* = 0.035) in nerve injury (*n* = 9) and sham (*n* = 3) samples in the GSE102721 dataset. (C) The expression of *Atf3* (^****^ *p* = 6.7e‐05), *Bin2* (^**^ *p* = 0.00147), *Fcgr2b* (^**^ *p* = 0.00234), *Nlrp3* (^***^ *p* = 0.00036), *Siglec8*, *Ucn* in nerve injury (*n* = 15) and sham (*n* = 3) samples in the GSE175760 dataset. *p*‐values were calculated using the Wilcoxon rank‐sum test to compare the two groups (nerve injury vs. sham). (D) The RAU value of genes in the training cohort. (E)–(F) The RAU value of genes in the GSE102721 and GSE175760 validation set.

Based on the expression levels observed for hub genes related to nerve injury in the training cohort and the validation datasets GSE102721 and GSE175760. We discovered a notable increase in the expression of *Atf3*, *Bin2*, *Fcgr2b*, *Nlrp3*, *Reg3a*, *Siglec8*, and *Ucn* in nerve injury samples when compared to sham samples in the training cohort (Figure 5A). Within the GSE102721 dataset, a significant upregulation of *Atf3*, *Fcgr2b*, and *Ucn* was recorded in nerve injury samples (Figure 5B; nerve injury vs. sham). In the GSE175760 dataset, the expressions of *Atf3*, *Bin2*, *Fcgr2b*, and *Nlrp3* showed significant elevation in nerve injury samples relative to sham samples (Figure 5C). The ROC curve analysis indicated that the AUC for all seven hub genes linked to nerve injury in the training cohort exceeded 0.7 (Figure 5D). Similarly, in the validation sets GSE102721 and GSE175760, the AUC values for *Atf3*, *Bin2*, *Fcgr2b*, and *Ucn* also surpassed 0.7 (Figure 5E,F). Consequently, *Atf3*, *Bin2*, *Fcgr2b*, and *Ucn* may serve as reliable biomarkers for nerve injury diagnosis and demonstrate significant diagnostic potential. Thus, we selected *Atf3*, *Bin2*, *Fcgr2b*, and *Ucn* for the subsequent analysis.

In the training cohort, we assessed the expression correlations among *Atf3*, *Bin2*, *Fcgr2b*, and *Ucn* (Figure S2A). Our analysis revealed that *Atf3* expression was inversely correlated with that of *Bin2*, *Ucn*, and *Fcgr2b*. Furthermore, *Fcgr2b* showed a positive correlation with *Bin2*, but a negative correlation with *Ucn*. Conversely, *Ucn* expression was negatively associated with *Bin2* (Figure S2A). Moreover, we predicted several specific miRNA‐mRNA interactions: both *Bin2* and *Ucn* were identified as potential targets of miR‐344b‐5p and miR‐483‐3p; *Bin2* and *Atf3* were predicted to be targeted by miR‐149‐3p; and *Atf3* and *Fcgr2b* were identified as potential targets of miR‐25‐5p (Figure S2B).

### 3.5 GSEA Enrichment Analysis

**FIGURE 6:**
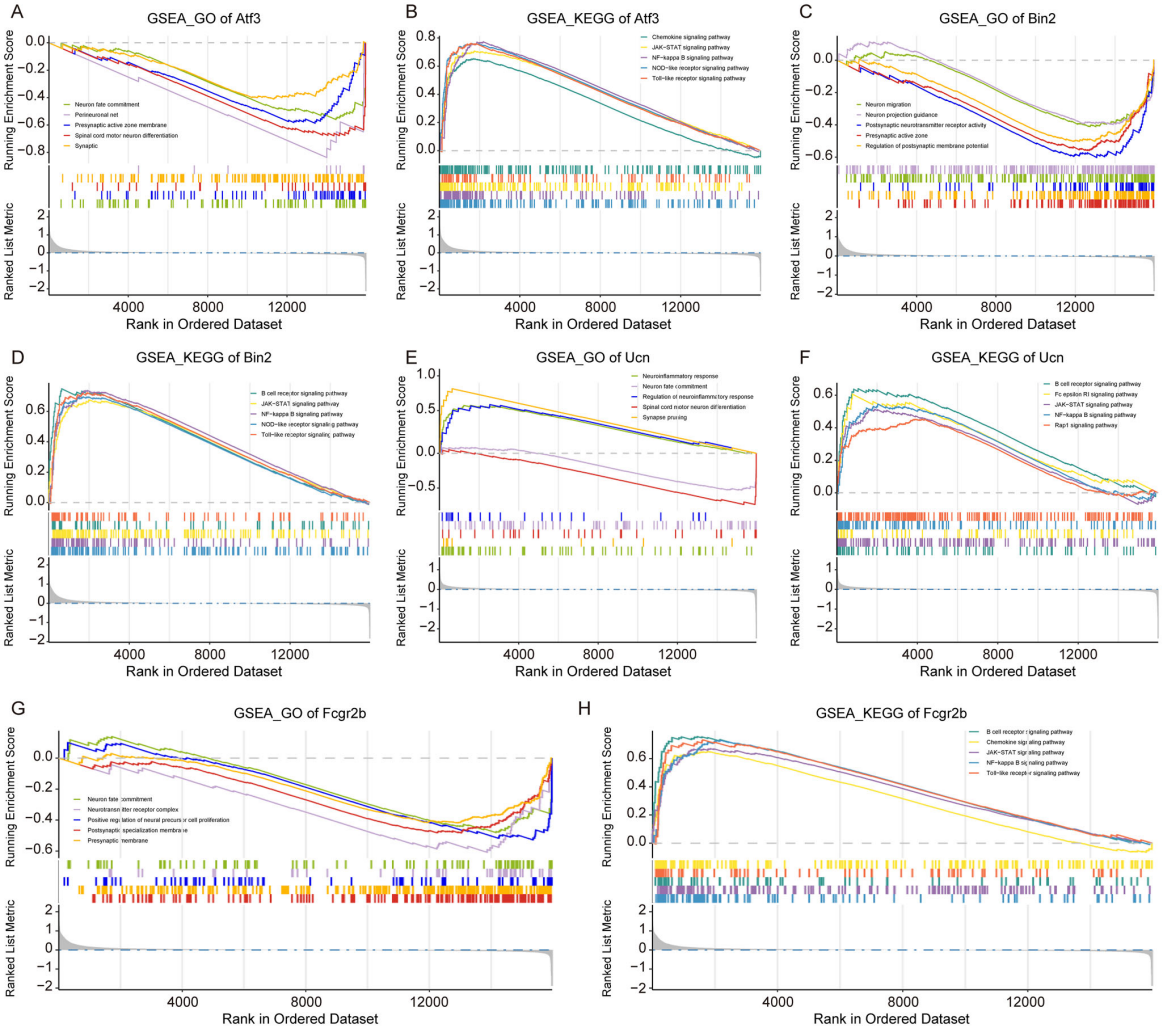
GO and KEGG enrichment analysis based on GSEA. (A)–(B) Enrichment plots depicted of *Atf3* by gene set enrichment analysis (GSEA) based on GO (A) and KEGG (B) gene sets. (C)–(D) Enrichment plots depicted of *Bin2* by gene set enrichment analysis (GSEA) based on GO (C) and KEGG (D) gene sets. (E)–(F) Enrichment plots depicted of *Ucn* by gene set enrichment analysis (GSEA) based on GO (E) and KEGG (F) gene sets. (G)–(H) Enrichment plots depicted of *Fcgr2b* by gene set enrichment analysis (GSEA) based on GO (G) and KEGG (H) gene sets.

Furthermore, we explored the potential biological functions of *Atf3*, *Bin2*, *Fcgr2b*, and *Ucn* using GSEA analysis. In the training cohort, GSEA was conducted to evaluate the GO and KEGG enrichment for the high and low expression groups of each gene (*Ucn*, *Atf3*, *Fcgr2b*, *Bin2*) (Table S5). The results showed that *Atf3* was mainly associated with biological functions of neuronal fate commitment, perineuronal net, and presynaptic active zone membrane dynamics. It was correlated with chemokine signaling pathway, JAK‐STAT signaling pathway, and NF‐kappa B signaling pathway (Figure 6A,B). *Bin2* was mainly associated with neuron migration, neuron projection guidance, and postsynaptic neurotransmitter receptor activity, biological functions, and was associated with B‐cell receptor signaling pathway, JAK‐STAT signaling pathway, and NF‐kappa B signaling pathway (Figure 6C,D). *Ucn* was mainly involved in the neuroinflammatory response, neuronal fate commitment, and regulation of neuroinflammatory response, and was associated with the B cell receptor pathway, Fc epsilon RI pathway, and JAK‐STAT pathway (Figure 6E,F). *Fcgr2b* was mainly associated with neuronal fate commitment, neurotransmitter receptor complex, and positive regulation of neural precursor cell proliferation, biological processes, and was linked to the B cell receptor signaling pathway, chemokine signaling pathway, and JAK‐STAT signaling pathway (Figure 6G,H). These results indicate that *Ucn*, *Atf3*, *Fcgr2b*, and *Bin2*are critically involved in inflammation and immune responses in nerve injury.

### 3.6 The Correlation Between *Ucn*, *Atf3*, *Fcgr2b* and *Bin2* Genes and the Infiltration of Immune Cells in Nerve Injury

**FIGURE 7:**
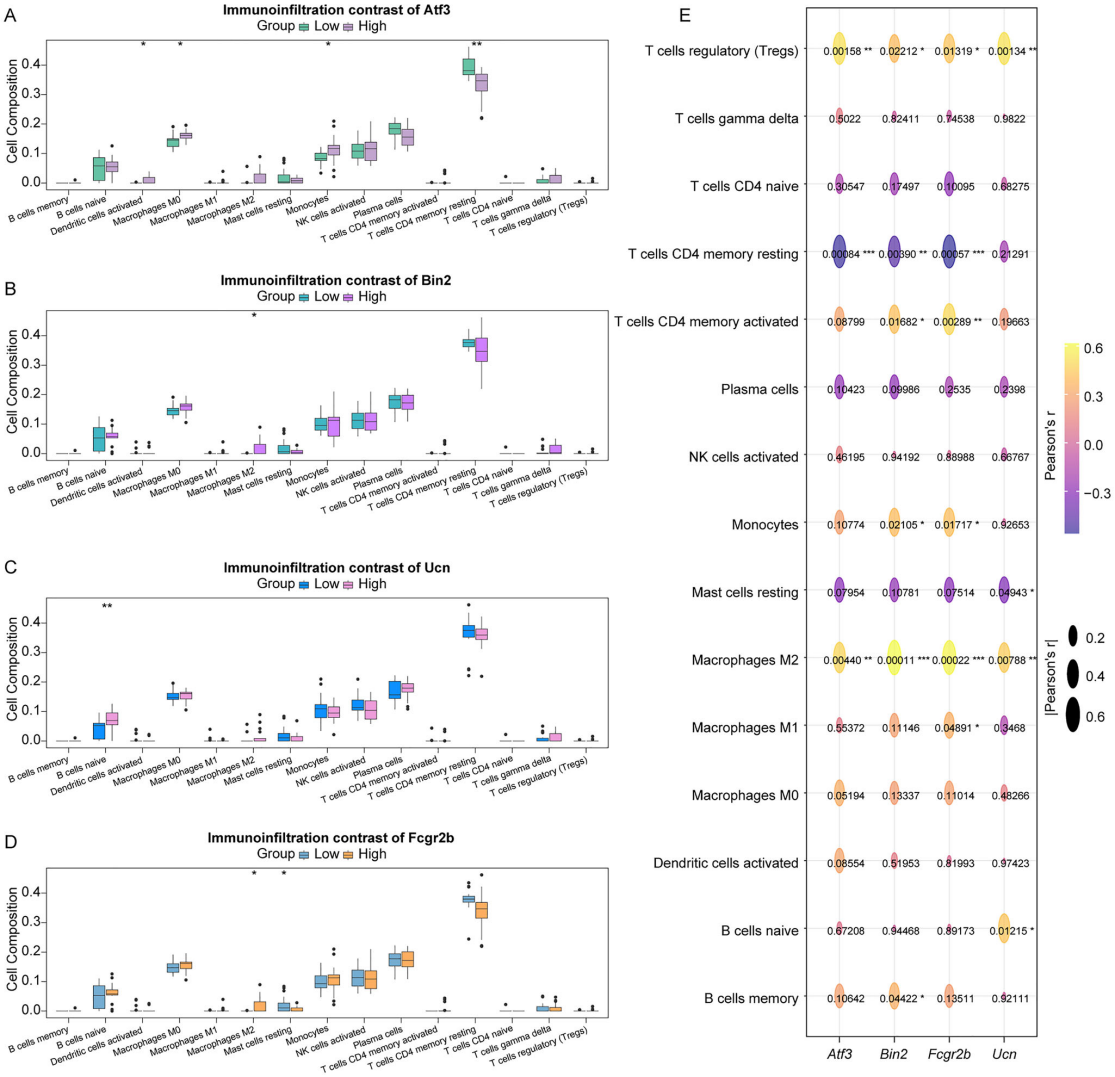
Immune cell infiltration. (A) The proportion of immune cell infiltration in the high (*n* = 17) and low (*n* = 17) *Atf3* expression groups in the training cohort (activated dendritic cells: ^*^ *p* = 0.0296; macrophages M0: ^*^ *p* = 0.0106; monocytes: ^*^ *p* = 0.0235; resting memory CD4+ T cells: ^**^ *p* = 0.0011). (B) The proportion of immune cell infiltration in the high (*n* = 17) and low (*n* = 17) *Bin2* expression groups in the training cohort (macrophages M2: ^*^ *p* = 0.015). (C) The proportion of immune cell infiltration in the high (*n* = 17) and low (*n* = 17) *Ucn* expression groups in the training cohort (naïve B cells: ^**^ *p* = 0.0067). (D) The proportion of immune cell infiltration in the high (*n* = 17) and low (*n* = 17) *Fcgr2b* expression groups in the training cohort (macrophages M2: ^*^ *p* = 0.015; resting mast cells: ^*^ *p* = 0.032). *p*‐values were calculated using the Wilcoxon rank‐sum test to compare the two groups (high vs. low). (E) The correlation of *Ucn*, *Atf3*, *Fcgr2b*, and *Bin2* with immune cell infiltration.

In the training cohort, we calculated the infiltration of immune cells in nerve injury samples using CIBERSORT algorithm. Subsequently, we analyzed the differences in immune cell infiltration between the high and low expression groups of each gene (*Ucn*, *Atf3*, *Fcgr2b*, *Bin2*). As illustrated in Figure 7A, the high *Atf3* expression group demonstrated a greater proportion of activated dendritic cells, M0 macrophages, and monocytes, alongside a reduced proportion of resting memory CD4+ T cells (high vs. low; *p* < 0.05). Furthermore, the proportions of M2 macrophages and naïve B cells were significantly elevated in the high *Bin2* and high *Ucn* expression groups (Figure 7B,C, high vs. low; *p* < 0.05), respectively. Additionally, the proportion of M2 macrophages was significantly increased, whereas the proportion of resting mast cells was markedly decreased in the high *Fcgr2b* expression group (Figure 7D, high vs. low; *p* < 0.05). Correlation analysis revealed a significant positive correlation between the expression levels of *Ucn*, *Atf3*, *Fcgr2b*, and *Bin2* with the proportion of M2 macrophages (Figure 7E). Additionally, *Atf3*, *Fcgr2b*, and *Bin2* expression demonstrated a significant negative correlation with resting memory CD4+ T cells (Figure 7E).

### 3.7 Cell‐Specific Expression Patterns of *Ucn*, *Atf3*, *Fcgr2b*, and *Bin2* in Neural Cells

**FIGURE 8:**
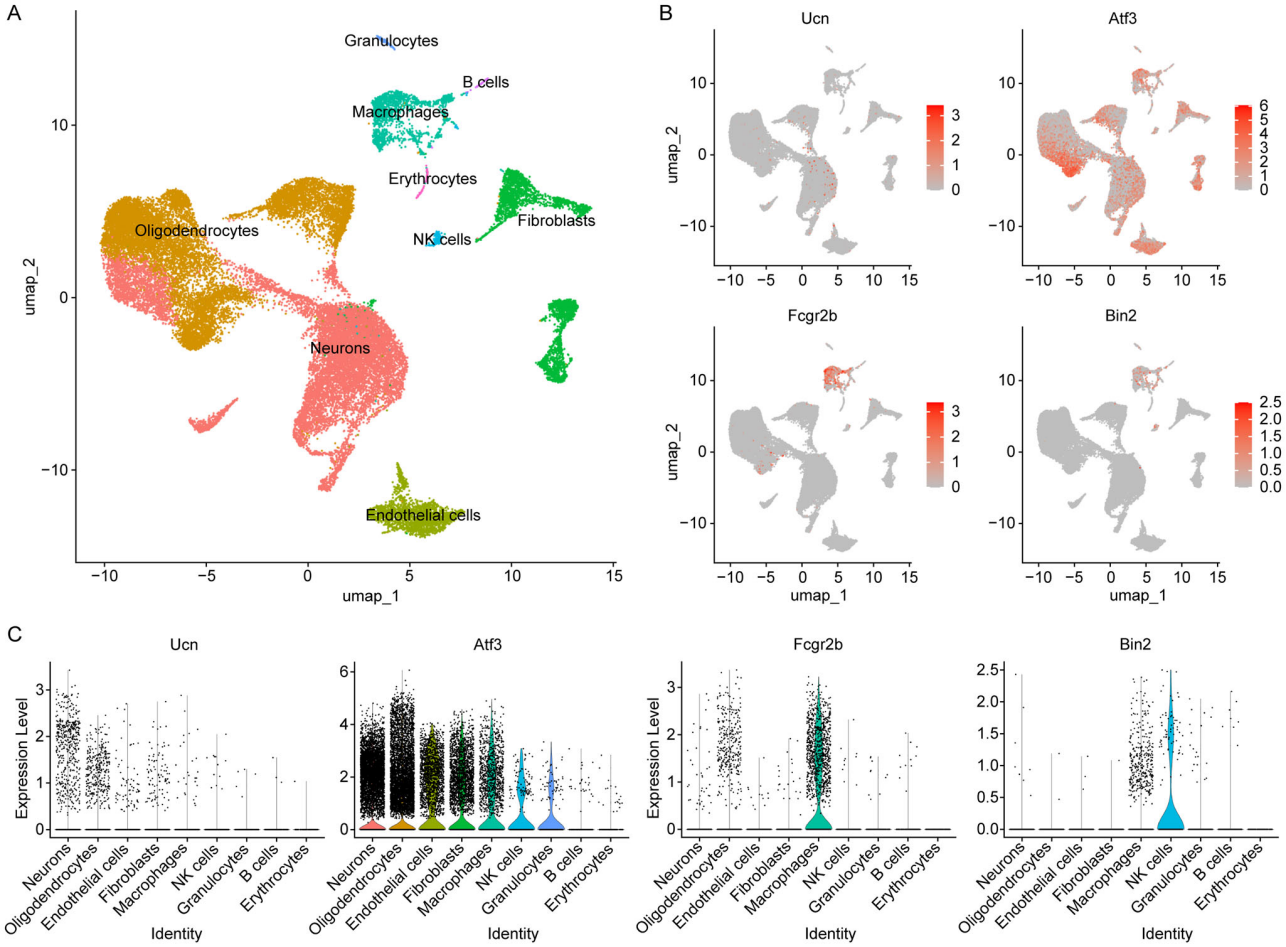
Cell‐specific expression patterns of *Ucn*, *Atf3*, *Fcgr2b*, and *Bin2* in neural cells. (A) Cell Subpopulation Annotation Map of Dorsal Root Ganglion Tissue. (B) Expression patterns of *Ucn*, *Atf3*, *Fcgr2b*, and *Bin2* in different cell subtypes. (C) The expression levels of *Ucn*, *Atf3*, *Fcgr2b*, and *Bin2* in different cell subtypes.

To investigate the expression patterns of *Ucn*, *Atf3*, *Fcgr2b*, and *Bin2* in neural cells, we conducted quality control, normalization, and batch effect removal on the scRNA data from the GSE255014 dataset. Subsequently, the dorsal root ganglion tissue was classified into nine distinct cell clusters: B cells, endothelial cells, erythrocytes, fibroblasts, granulocytes, macrophages, neurons, NK cells, and oligodendrocytes (Figure 8A). Subsequently, we analyzed the expression levels of four key genes within these cell populations. The results revealed that *Atf3* and *Ucn* were predominantly expressed at high levels in neurons, oligodendrocytes, and other cells associated with the nervous system (Figure 8B,C). In contrast, *Fcgr2b* and *Bin2* exhibited significantly higher expression levels in macrophages, NK cells, and other immune‐related cells (Figure 8B,C).

### 3.8 *Fcgr2b* Correlated With Neurite Outgrowth of PC12 Cells

**FIGURE 9:**
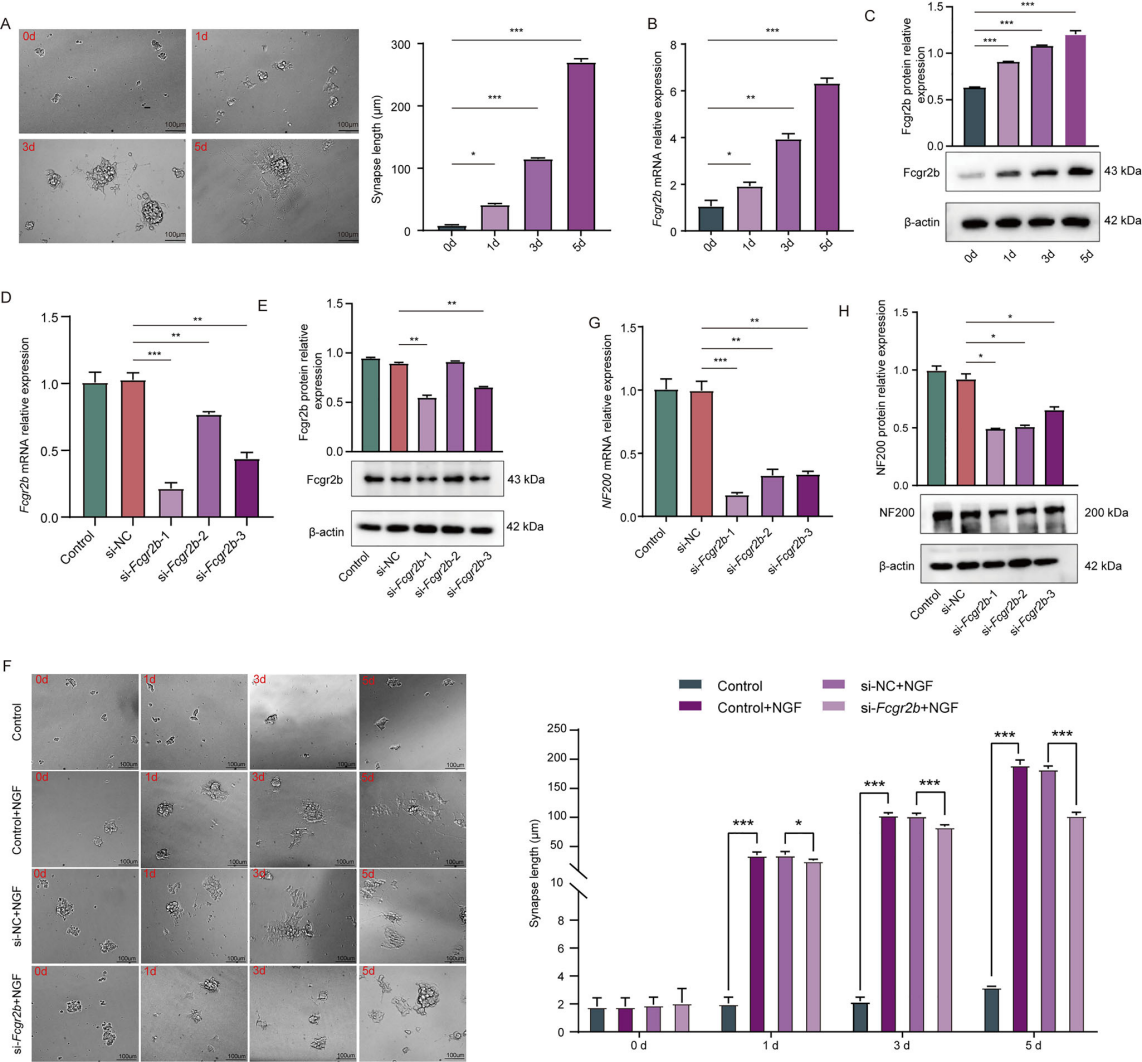
*Fcgr2b* correlated with neurite outgrowth of PC12 Cells. (A) PC12 cells were treated with NGF over a period of 0 to 5 days. ^*^ *p*  =  0.0103 (unpaired *t*‐test, two‐tailed) (1 day vs. 0 day); ^***^ *p* = 0.0001 (Unpaired *t*‐test, two‐tailed) (3 day vs. 0 day); ^***^ *p* = 0.0005 (unpaired *t*‐test, two‐tailed) (5 day vs. 0 day). (B) The expression of *Fcgr2b* was found to increase, as evidenced by RT‐qPCR. ^*^ *p*  =  0.048 (unpaired *t*‐test, two‐tailed) (1 day vs. 0 day); ^**^ *p* = 0.001053 (unpaired *t*‐test, two‐tailed) (3 day vs. 0 day); ^***^ *p* = 0.000098 (unpaired *t*‐test, two‐tailed) (5 day vs. 0 day). (C) The expression of Fcgr2b was found to increase, as evidenced by and western blot analyses. ^***^ *p* <0.0001 (unpaired *t*‐test, two‐tailed) (1 day vs. 0 day; 3 day vs. 0 day; 5 day vs. 0 day). (D) The knockdown efficiency of *Fcgr2b* was confirmed through RT‐qPCR assay. ^***^ *p* = 0.000274 (unpaired *t*‐test, two‐tailed) (si‐*Fcgr2b*‐1 vs. si‐NC); ^**^ *p* = 0.009853 (Unpaired *t*‐test, two‐tailed) (si‐*Fcgr2b*‐2 vs. si‐NC); ^**^ *p* = 0.001042 (unpaired *t*‐test, two‐tailed) (si‐*Fcgr2b*‐3 vs. si‐NC). (E) The knockdown efficiency of *Fcgr2b* was confirmed through western blotting. ^**^ *p* = 0.0014 (unpaired *t*‐test, two‐tailed) (si‐*Fcgr2b*‐1 vs. si‐NC); ^**^ *p* = 0.0033 (unpaired *t*‐test, two‐tailed) (si‐*Fcgr2b*‐3 vs. si‐NC). (F) The average neurite lengths of siRNA‐NC and si‐*Fcgr2b* transfected PC12 cells. ^***^ *p*<0.0001 (Unpaired *t*‐test, two‐tailed) (Control+NGF vs. Control, 1d); ^*^ *p* = 0.0163 (Unpaired *t*‐test, two‐tailed) (si‐*Fcgr2b*+NGF vs. si‐NC+NGF, 1d); ^***^ *p*<0.0001 unpaired *t*‐test, two‐tailed) (Control+NGF vs. Control, 3d and 5d); ^***^ *p*<0.0001 (Unpaired *t*‐test, two‐tailed) (si‐*Fcgr2b*+NGF vs. si‐NC+NGF, 3d and 5d). (G) The expression of NF200 in siRNA‐NC and si‐*Fcgr2b* transfected PC12 cells was determined via RT‐qPCR assay. ^***^ *p* = 0.000431 (unpaired *t*‐test, two‐tailed) (si‐*Fcgr2b*‐1 vs. si‐NC); ^**^ *p* = 0.001661 (unpaired *t*‐test, two‐tailed) (si‐*Fcgr2b*‐2 vs. si‐NC); ^**^ *p* = 0.001085 (unpaired *t*‐test, two‐tailed) (si‐*Fcgr2b*‐3 vs. si‐NC). (H) The expression of NF200 in siRNA‐NC and si‐*Fcgr2b* transfected PC12 cells was determined via western blot assays. ^*^ *p* = 0.0253 (unpaired *t*‐test, two‐tailed) (si‐*Fcgr2b*‐1 vs. si‐NC); ^*^ *p* = 0.0194 (unpaired *t*‐test, two‐tailed) (si‐*Fcgr2b*‐2 vs. si‐NC); ^*^ *p* = 0.0347 (unpaired *t*‐test, two‐tailed) (si‐*Fcgr2b*‐3 vs. si‐NC). Each data point is presented as the mean± SE. All experiments were performed in three biological replicates (*n* = 3).

Finally, we investigated the role of *Fcgr2*b in neuronal outgrowth following nerve injury. NGF at a concentration of 100 ng/mL was employed to stimulate the outgrowth of naive PC12 cells. Morphological analysis demonstrated that PC12 cells developed extensive neurites after treatment over a period of 0 to 5 days (Figure 9A). During this period, the expression of *Fcgr2b* was found to increase, as evidenced by RT‐qPCR and western blot analyses (Figure 9B,C). Subsequently, we knocked down the expression of *Fcgr2b* in PC12 cells to investigate the relationship between *Fcgr2b* and neurite outgrowth. The knockdown efficiency of *Fcgr2b* was confirmed through RT‐qPCR and western blotting (Figure 9D,E). Following the reduction of *Fcgr2b* expression, neurite outgrowth was significantly inhibited, as indicated by the decreased length of neurites in PC12 cells stimulated with NGF for 1, 3, and 5 days (Figure 9F). Additionally, we observed a reduction in the expression of NF200, which was closely associated with neurite outgrowth (Figure 9G,H).

## 4 Discussion

Nerve injury is a prevalent, disabling condition encountered in clinical practice, resulting from various factors such as mechanical trauma, crush injuries, or iatrogenic damage (Lavorato et al. 2023; Robinson 2022). These injuries often lead to severe impairments in sensory, motor, and autonomic nerve functions in affected patients (Bhandari 2019; Robinson 2022). Despite significant advancements in nerve regeneration research in recent years, many patients continue to experience long‐term functional deficits due to limited axonal regenerative capacity, inhibitory microenvironmental factors, and inadequate neurotrophic support (Lam and Leung 2024; Tian et al. 2025). Research has demonstrated that dysregulated inflammatory responses following trauma, overactivation of glial cells, and abnormal remodeling of the extracellular matrix are critical factors that impede nerve regeneration and repair (Bonnans et al. 2014; Golshadi et al. 2023). Consequently, a thorough analysis of the pathological mechanisms following nerve injury, along with the precise identification of key regulatory markers, is crucial for the development of innovative rehabilitation treatment strategies. In this study, we identified *Atf3*, *Bin2*, *Fcgr2b*, and *Ucn* as critical biomarkers of traumatic nerve injury by integrating bioinformatics and machine learning techniques. These biomarkers played important roles in neuroinflammation, immune modulation, and neural repair. In addition, their cell‐specific expression patterns and pathway associations provided novel insights into therapeutic targeting for nerve regeneration.

A total of 42 genes were identified as differentially expressed between nerve injury samples and sham surgery samples, correlating with the onset of nerve injury. Enrichment analysis revealed that these 42 genes were significantly associated with immune‐related and infection/inflammatory pathways. These findings suggest that immune dysregulation and inflammatory responses play a pivotal role in the pathogenesis of nerve injury, aligning with previous studies that emphasize the detrimental effects of chronic inflammation on neural repair (Bray et al. 2022; Xu et al. 2025). To refine our candidate gene list, we utilized three distinct machine learning algorithms—LASSO, SVM‐RFE, and Random Forest—which collectively prioritized seven high‐confidence genes related to nerve injury. Among these, *Atf3*, *Bin2*, *Fcgr2b*, and *Ucn* consistently exhibited significant upregulation in nerve injury samples across both the training cohort and independent validation datasets. Notably, ROC curve analysis demonstrated robust diagnostic potential for these genes, with AUC values exceeding 0.7 in all tested cohorts. This high predictive accuracy underscores their utility as potential biomarkers for the diagnosis and monitoring of nerve injury progression.

The observed co‐expression patterns among Atf3, Bin2, Fcgr2b, and Ucn suggested potential functional interplay beyond simple correlation. The inverse correlation between *Atf3* and *Bin2* was particularly noteworthy. Given that Atf3 is a stress‐inducible transcription factor known to repress target genes by recruiting HDAC1 to their promoters (Saha et al. 2021), we hypothesized that *Atf3* might act as an upstream transcriptional repressor of *Bin2*. This hypothesis was supported by the predicted miRNA‐mRNA network, which indicated that both *Bin2* and *Atf3* were targeted by miR‐149‐3p, suggesting their possible co‐regulation within a shared post‐transcriptional network. Furthermore, the prediction that both *Atf3* and *Fcgr2b* were potential targets of miR‐25‐5p provided a compelling mechanistic basis for their coordinated expression. miR‐25‐5p has been reported to function as an oncogenic miRNA in certain contexts and was implicated in regulating immune and inflammatory responses (Wang et al. 2022). Its simultaneous targeting of a transcription factor (*Atf3*) and an immunoreceptor (*Fcgr2b*) suggested that miR‐25‐5p could act as a master regulator coordinating gene expression networks across functional modules. Similarly, the predicted targeting of both *Bin2* and *Ucn* by miR‐344b‐5p and miR‐483‐3p further supported a plausible mechanistic link for their coordinated expression. A positive correlation was observed between *Fcgr2b* and *Bin2*, suggesting their potential cooperation within a shared pathway. Furthermore, functional enrichment analysis revealed that both genes were implicated in neuroinflammation and neuronal fate commitment pathways, reinforcing this functional coherence. Thus, experimental validation of these predicted miRNA‐mRNA interactions will be essential to fully elucidate this complex post‐transcriptional regulatory network.

The GSEA results indicated that the identified hub genes (*Ucn*, *Atf3*, *Fcgr2b*, and *Bin2*) were functionally enriched in neuroinflammation, neuronal fate commitment, and JAK‐STAT/NF‐κB signaling pathways. This suggests that these genes may play significant roles in the processes of nerve injury and repair. *Atf3*, a stress‐responsive transcription factor, is highly conserved within the vertebrate nervous system and plays a critical role in the early response to nerve injury (Katz et al. 2022). Research indicates that *Atf3* expression significantly increases following nerve injury, promoting neuronal survival and axon regeneration by regulating downstream gene expression (Petrovic et al. 2022). This regulatory mechanism is essential for functional recovery after nerve injury, underscoring the pro‐regenerative role of *Atf3* in neural repair processes. Ucn, a neuropeptide with anti‐inflammatory properties, serves a dual role in the repair process post‐nerve injury by exerting neuroprotective effects and modulating immune responses, thereby preventing excessive immune activation that could further damage neural tissues (Hakim et al. 2024; Wheeler and Quintana 2025). This dual mechanism endows *Ucn* with significant regulatory functions in the post‐injury repair process, aiding in balancing neuroprotection and immune activation. We observed that *Atf3* and *Ucn* were predominantly expressed at high levels in neurons, oligodendrocytes, and other cells associated with the nervous system. Moreover, *Atf3* and *Ucn* exhibited a positive correlation with the proportion of M2 macrophages in the context of nerve injury. These results indicate that *Atf3* and *Ucn* may facilitate neural repair by modulating the immune microenvironment. These findings suggest potential therapeutic targets for treatment following nerve injury; however, the specific regulatory mechanisms warrant further investigation.

The immune response plays a crucial role in the process of repair after neural injuries. The receptor FcγRIIB (*Fcgr2b*) is the only Fcγ receptor that serves an inhibitory purpose; its intracellular domain possesses an immunoreceptor tyrosine‐based inhibitory motif (ITIM). When it interacts with immune complexes, *Fcgr2b* experiences phosphorylation of the ITIM, which subsequently hampers signaling pathways related to cell activation. This inhibitory influence is evident in a variety of immune cells, such as B cells and myeloid cells, effectively reducing excessive activation of the immune system and inflammatory responses (Smith and Clatworthy 2010). Additionally, a lack of sufficient *Fcgr2b* expression on B cells is closely linked to the development of chronic inflammatory demyelinating polyneuropathy (CIDP) (Tackenberg et al. 2009). This indicates that *Fcgr2b* plays an essential role in controlling B cell activation and reducing inflammation; its dysfunction may result in heightened inflammatory responses following nerve damage. Other research has demonstrated that elevated levels of *Fcgr2b* are closely associated with the infiltration of immune cells and the expression of immune checkpoint molecules within the glioma tumor microenvironment (Sun et al. 2023). Our study revealed a positive correlation between *Fcgr2b* and macrophage M2 in nerve injury samples, with significant *Fcgr2b* expression in these cells. Moreover, Macrophages promote axonal regeneration synergistically by secreting growth factors (such as NGF, BDNF) and clearing inhibitory debris after nerve injury (Gensel et al. 2009). We discovered that *Fcgr2b* promoted the outgrowth of neurites in PC12 cells. Therefore, Fcgr2b may be a key regulatory factor connecting immunosuppression and neural regeneration, providing a potential therapeutic target for the recovery of nerve injury.

This study has several limitations that should be addressed in future research. First, all transcriptomic data and validation analyses were derived from rat models. While these models provide valuable insights, the conservation and translational relevance of the identified biomarkers—*Atf3*, *Bin2*, *Fcgr2b*, and *Ucn*—in human nerve injury remains to be confirmed. Second, the transcriptome data were obtained from a single post‐injury time point, which does not capture the dynamic expression patterns of these genes throughout the injury and regeneration process. Third, although we validated the role of Fcgr2b in neurite outgrowth using in vitro experiments, functional studies involving gain‐ or loss‐of‐function approaches for the other key genes (*Atf3*, *Bin2*, and *Ucn)* are still needed to fully elucidate their mechanisms. Finally, the diagnostic and therapeutic potential of these biomarkers requires further validation in human clinical samples, such as serum or nerve tissue from patients with peripheral or central nerve injuries. Future work should include multi‐timepoint transcriptional profiling, functional verification of all key genes, and correlation analysis with clinical phenotypes in human cohorts.

## 5 Conclusion

**FIGURE 10:**
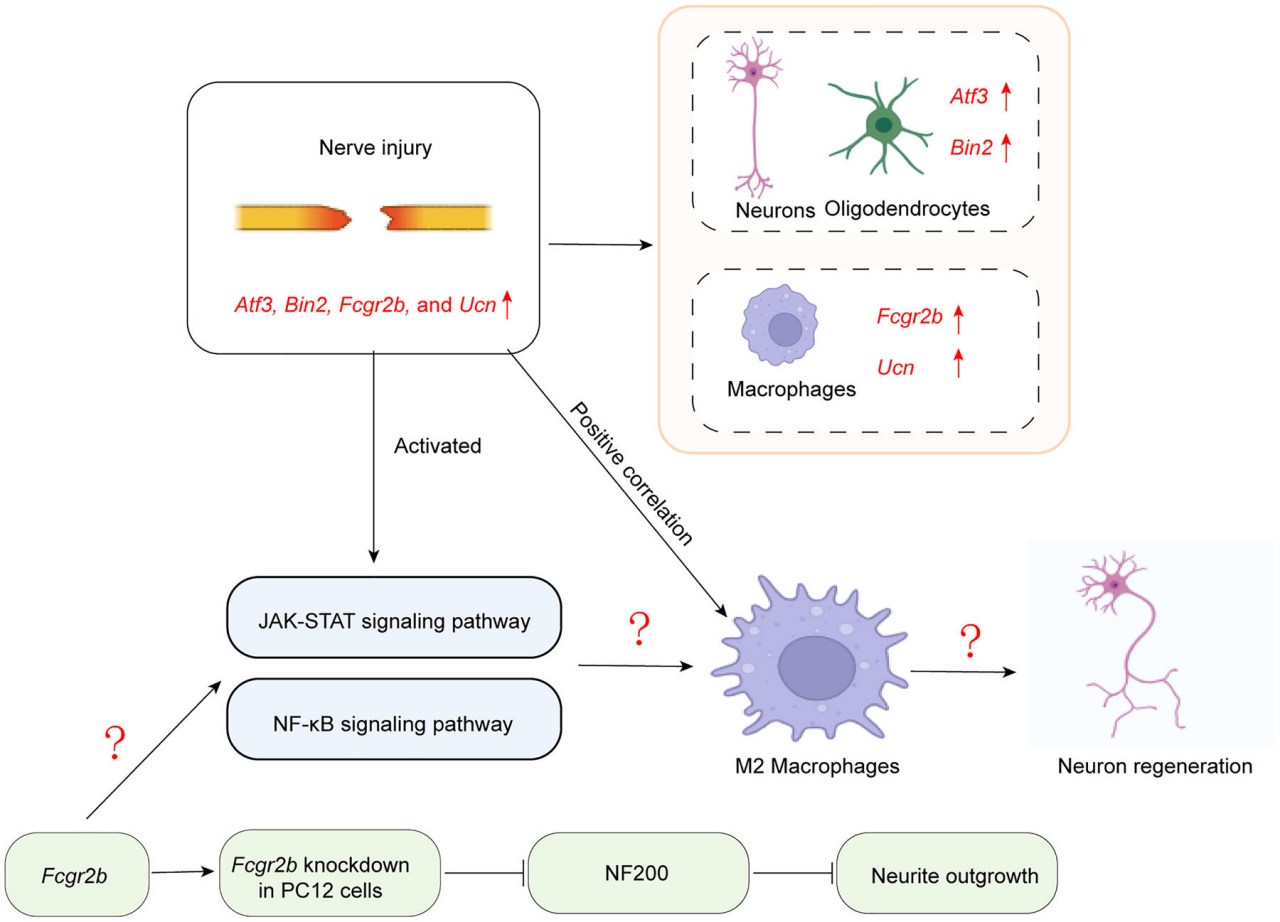
Schematic of the neuro‐immune crosstalk in nerve injury.

In conclusion, our study identified *Atf3*, *Bin2*, *Fcgr2b*, and *Ucn* as key genes associated with nerve injury, with potential diagnostic and therapeutic applications. These genes are implicated in the JAK‐STAT and NF‐κB signaling pathways. Single‐cell analysis revealed significant enrichment of *Atf3* and *Ucn* in neurons and oligodendrocytes, while *Fcgr2b* and *Bin2* were predominantly enriched in macrophages. Furthermore, the expression of all four genes showed a significant positive correlation with M2 macrophage polarization. Functional experiments demonstrated that knockdown of *Fcgr2b* inhibited neurite outgrowth in PC12 cells (Figure 10). However, it remains unclear whether *Fcgr2b* promotes nerve regeneration after injury by modulating M2 macrophage polarization through the JAK‐STAT or NF‐κB pathways.

## Author Contributions

Conceptualization: Shuming Cao and Chengyue Yu. Data curation: Shuming Cao and Chengyue Yu. Formal analysis: Shuming Cao and Chengyue Yu. Investigation: Shuming Cao and Chengyue Yu. Methodology: Shuming Cao and Chengyue Yu. Writing – original draft:: Shuming Cao. Software: Nana Wang and Jianhua Xu. Validation: Nana Wang and Jianhua Xu. Visualization: Nana Wang and Jianhua Xu. Project administration: Weiguo Xu. Resources: Weiguo Xu. Supervision: Weiguo Xu. Writing – review & editing: Weiguo Xu. All authors read and approved the final version to be published.

## Funding

The authors have nothing to report.

## Conflicts of Interest

The authors declare no conflicts of interest.

## Supporting information




**Figure S1** Principal component analysis (PCA) plots with or without the elimination of batch effects. A. PCA plot without batch effect elimination. B. PCA plot with batch effect elimination. C. The expression levels of samples without the elimination of batch effects in the training cohort. D. The expression levels of samples with the elimination of batch effects in the training cohort.

**Figure S2** Analysis of gene correlations and predicted miRNA‐mRNA interactions in the training cohort. A. Expression correlation analysis among *Atf3*, *Bin*2, *Fcgr2b*, and *Ucn*. B. Predicted miRNA‐target gene interactions.

**Table S1** The genes in the turquoise module

**Table S2** Differentially expressed genes between the nerve injury and sham surgery groups

**Table S3** The results of GO and KEGG analysis

**Table S4** Hub nerve injury‐related genes were identified via three machine learning algorithms

**Table S5** The results of gene set enrichment analysis

## Data Availability

The data that support the findings of this study are openly available in Gene Expression Omnibus (GEO) at https://www.ncbi.nlm.nih.gov/geo/, reference numbers GSE236754, GSE202111, GSE172133, GSE102721, GSE175760, and GSE255014.
