# Supplementary figures and images for: Screening Biomarkers for Nerve Injury Using Weighted Gene Co‐Expression Network Analysis and Machine Learning
Source: Brain Behav. 2026 Feb 24;16(2):e71279. doi: 10.1002/brb3.71279 (PMC12931491; doi:10.1002/brb3.71279)

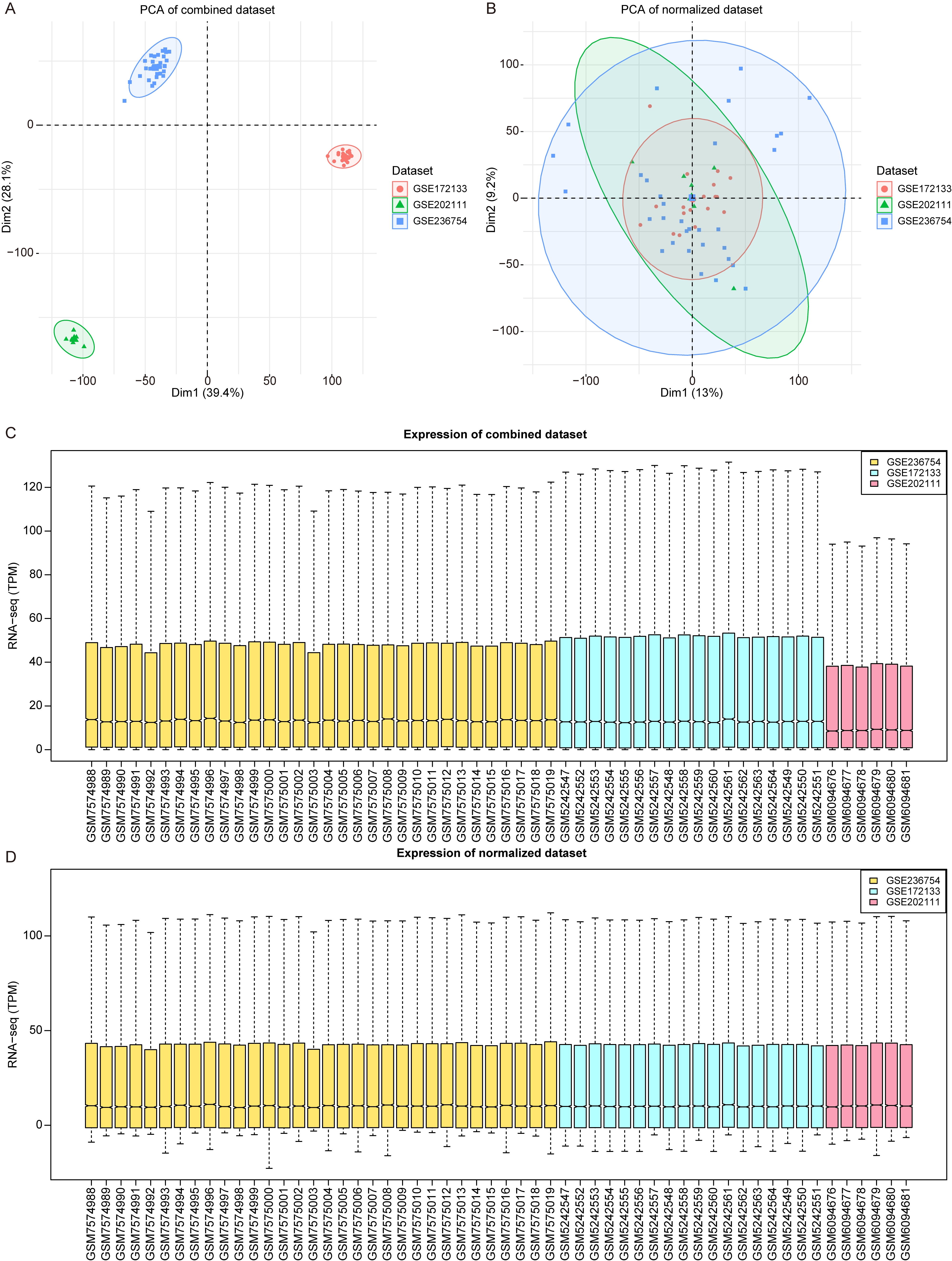

Supplement: Supplementary file 1 — Figure S1 Principal component analysis (PCA) plots with or without the elimination of batch effects. A. PCA plot without batch effect elimination. B. PCA plot with batch effect elimination. C. The expression levels of samples without the elimination of batch effects in the training cohort. D. The expression levels of samples with the elimination of batch effects in the training cohort. [file BRB3-16-e71279-s005.tif]

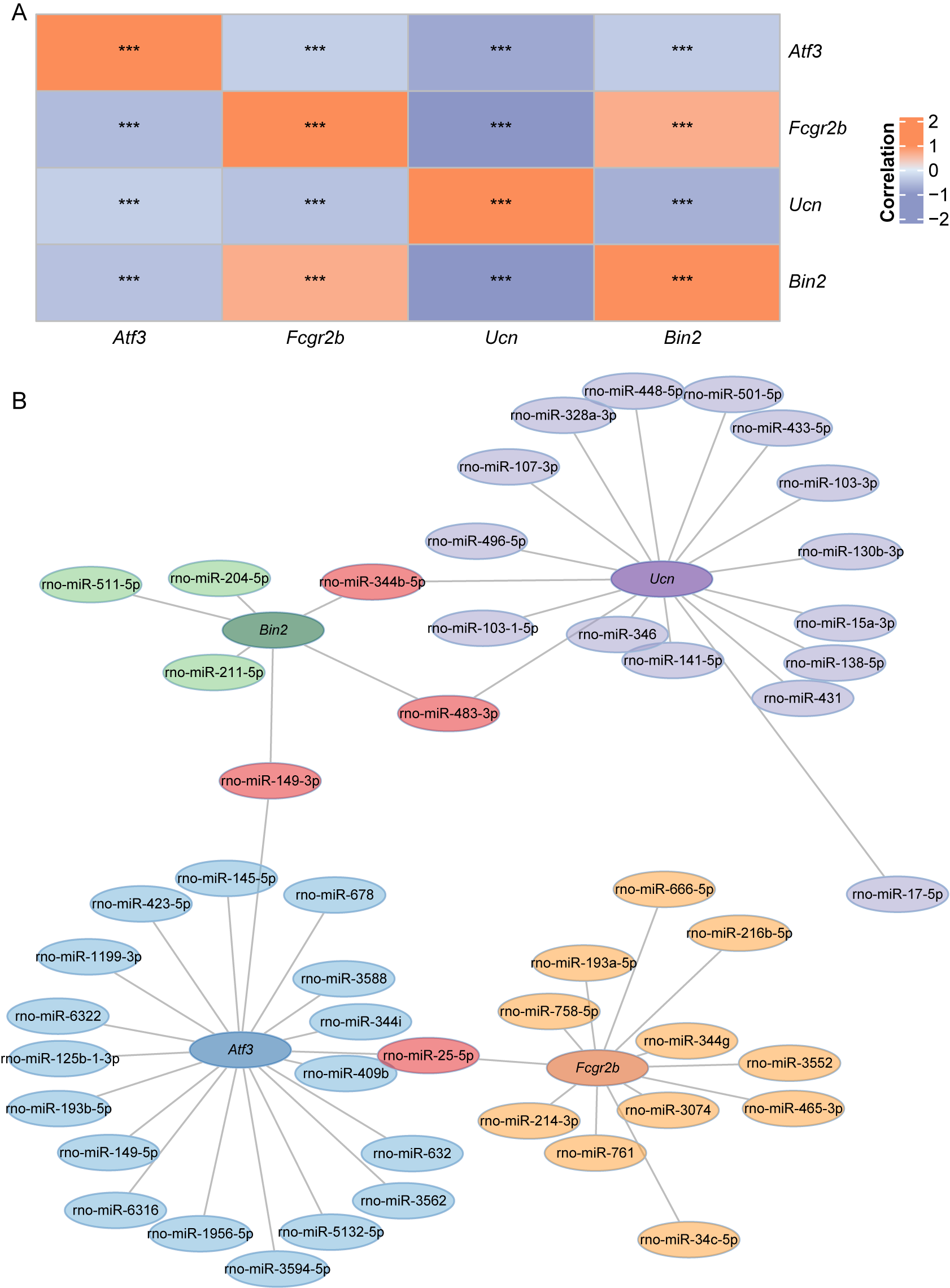

Supplement: Supplementary file 2 — Figure S2 Analysis of gene correlations and predicted miRNA‐mRNA interactions in the training cohort. A. Expression correlation analysis among Atf3, Bin2, Fcgr2b, and Ucn. B. Predicted miRNA‐target gene interactions. [file BRB3-16-e71279-s006.tif]
